# Characterization of the complete mitochondrial genome sequence of *Asiadodis yunnanensi*s (Mantidae: Choeradodinae) and phylogenetic analysis

**DOI:** 10.1080/23802359.2019.1660250

**Published:** 2019-09-02

**Authors:** Yan Shi, Qin-Peng Liu, Lan Luo, Zhong-Lin Yuan

**Affiliations:** Key Lab of Integrated Crop Pest Management of Shandong Province, College of Plant Health and Medicine, Qingdao Agricultural University, Qingdao, China

**Keywords:** *Asiadodis yunnanensis*, praying mantises, mitogenome

## Abstract

The complete mitochondrial genome of the praying mantises *Asiadodis yunnanensis* was characterized in this study. The circular molecule is 15,416 bp in length (GenBank accession no. MN037794), containing 13 protein-coding genes (PCGs), two ribosomal RNA (rRNA) genes, 22 transfer RNA (tRNA) genes. The nucleotide composition is asymmetric (39.5% A, 15.1% C, 9.6% G, 35.8% T), with an overall A + T content of 75.3%. The gene arrangement of *A. yunnanensis* is identical to that observed in other praying mantises. Seven PCGs are initiated with typical ATN start codons, Four genes (*cox1*, nad3, nad4L, and *nad6*) begin with TTN, nad1 begin with GTT, and *nad5* use CTT, as initiation codon. Twelve PCGs stop with complete termination codon TAA and TAG, whereas *nad5* uses incomplete termination codon (T––). Twenty reading frame overlaps and seven intergenic regions are found in the mitogenome of *A. yunnanensis*. The phylogenetic relationships based on 13 PCGs show that *A. yunnanensis* clusters closest to the species of Mantidae.

*Asiadodis* was established by Roy (2004) to distinguish from Choeradodis. *Asiadodis* have only been found in Southeast Asia and South Asia. There are two species involved in: *A. yunnanensis* and *A. squilla*. *A. yunnanensis* (Wang and Liang 1995) is a type of praying mantises belonging to the order Mantodea, family Mantidae, subfamily Choeradodinae, genus *Asiadodis*, distributing in China, Yunnan. *Asiadodis yunnanensis* captures insect pests as food and is recognized as an important natural enemy for biological control. In this study, samples of *A. yunnanensis* were collected from China, Mengla, Xishuangbanna, Yunnan (21.47°N, 101.54°E) and stored in the praying mantises specimen room of College of Plant Medicine, Qingdao Agricultural University with an accession number 01-XXBNJH.

The complete mitogenome of *A. yunnanensis* is a circular molecule of 15,416 bp in length (GenBank under accession no. MN037794) and contains 13 protein-coding genes (PCGs), 22 transfer RNA genes (tRNAs), two ribosomal RNA genes (*rrnL* and *rrnS*). The nucleotide composition of *A. yunnanensis* mitogenome is asymmetric (39.5% A, 15.1% C, 9.6% G, 35.8% T), with an overall A + T content of 75.3%. The AT-skew and GC-skew of this genome were 0.049 and −0.223, respectively. The gene organization of *A. yunnanensis* is similar to that observed in other praying mantises (Ye et al. 2016; Zhang et al. 2018). Twenty-one genes were encoded on the major strand (J-strand), whereas the others were encoded on the minor strand (N-strand).

The *A. yunnanensis* mitogenome harbours a total of 32 bp intergenic spacer sequences, which is made up of 5 regions in the range from 5 to 10 bp. Gene overlaps were found at nine gene junctions and involved a total of 223 bp, the longest 39 bp overlapping located between *nad3* and *trnL2*. The A + T content of the *A. yunnanensis* mitogenome was 75.3%. The higher A + T content of *A. yunnanensis* was present in all regions, both genes and noncoding regions. Gln (Q), Glu (E), and Trp (W) are most frequently used. Moreover, the A + T content were also reflected further in the codon usage: the relative synonymous codon usages showed that *A. yunnanensis* used more NNA and NNT codon. The *rrnL* was 1313 bp in length with A + T content of 79.3%, and *rrnS* was 767 bp in length with A + T content of 74.9%.

Seven PCGs in *A. yunnanensis* mitogenome start with a typical ATN (ATA and ATG) codon, four genes begin with TTN (TTG, ATC, and TTA), *nad1* uses GTT and nad5 use CTT as initiation codon. Twelve PCGs stop with complete termination codon (TAA and TAG), whereas *nad5* uses incomplete codon (T––) as termination codon. Based on the Maximum-likelihood analyses, we constructed the phylogenetic relationships of *A. yunnanensis* and 23 other praying mantises based on the 13 PCGs amino acids using RAxML. *Cryptocercus kyebangensis* was used as an outgroup. The results have shown that *A. yunnanensis* is closely clustered with other species in family Mantidae (Figure 1), which agree with the morphology classifications.

**Figure 1.**
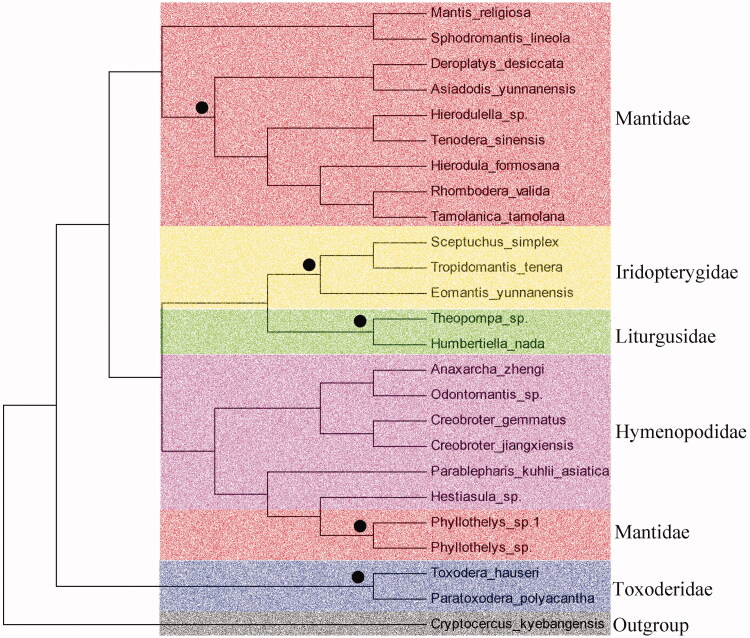
Phylogenetic tree showing the relationship between *A. yunnanensis* (MN037794) and 23 other praying mantises. *Cryptocercus kyebangensis* (KP872847.1) was used as an outgroup. The black circle indicates popularity >90. GenBank accession numbers used in the study are the following: *Rhombodera valida* (KX611804.1), *Hierodula formosana* (KR703238.1), *Tamolanica tamolana* (DQ241797.1), *Sphodromantis lineola* (KY689123.1), *Tenodera sinensis* (KY689132.1), *Hierodulella* sp. (KY689136.1), *Deroplatys desiccata* ( KY689113.1), *Mantis religiosa* (KU201317.1), *Sceptuchus simplex* (KY689133.1), *Tropidomantis tenera* (KY689127.1), *Eomantis yunnanensis* (KY689138.1), *Theopompa* sp. (KU201314.1), *Humbertiella nada* (KU201315.1), *Anaxarcha zhengi* (KU201320.1), *Odontomantis* sp. (KY689121.1), *Creobroter gemmatus* (KU201319.1), *Creobroter jiangxiensis* (KY689134.1), *Parablepharis kuhlii asiatica* (KY689117.1), *Hestiasula* sp. (KY689115.1), *Phyllothelys* sp.1 (KY689119.1), *Phyllothelys* sp.(KY689129.1), *Toxodera hauseri* (KX434837.1), and *Paratoxodera polyacantha* (MG049920.1). Mantis determined in this study was underlined.
